# Porcine skeletal muscle typing in histochemical and in-situ RT-PCR analysis

**DOI:** 10.1016/j.vas.2023.100332

**Published:** 2024-03-01

**Authors:** Tao Lin, Zhun Liu, Fawen Dai, Hechuan Wang, Jianjun Zuo

**Affiliations:** a College of Life Science, Leshan Normal University, Sichuan, 61400, China; b Key Laboratory of Bamboo Pest Control and Resource Development, Leshan, Sichuan 641000, People’s Republic of China; c South China Agricultural University, Guangdong, 510000, China; d Guang'an Academy of Agriculture and Forestry Sciences, China; e Guang'an Xinnong Development Co., Ltd, Guang'an, 638000, China

**Keywords:** Pigs, Muscle fiber classification, Enzyme histochemistry, In-situ RT-PCR

## Abstract

Currently, there are plenty of histochemical methods to classify pig muscle fibers, which confused the naming and classification of muscle fibers. This study aims to analyze the difference and correlation of 6 different histochemical methods and select the most suitable method for muscle fiber classification at the molecular and histomological levels by in-situ RT-PCR and enzyme histochemical methods. Muscle fiber samples, including psoas (PM), semitendinosus (SM) and trapezius muscle (TM), were collected from Large Spotted (LS), Lantang (LT) and Landrace (LR) pigs at their market-ages (LS at 150 d, LT at 210 d, and LR at 150 d). 6 kinds of histochemical methods combining actomyosin adenosine triphosphatase (AM-ATPase) with succinate dehydrogenase (SDH) enzyme were conducted to differentiate fiber types. 2 types of fibers (I and II) were differentiated by acid 2-fibre (2-AC) or alkaline 2-fibre classification(2-AL), 3 types of fibers (βR, αR and αW) by 3-AC or 3-AL, and 4 types of fibers (I, IIa, IIx and IIb) by 4-AC, or 4-AL. Results showed that AC and AL muscle-fiber classification were consistent in reflecting the characteristics of muscle fibers(*P* > 0.05), but the color of each muscle fiber type was just opposite. AC methods may be superior to AL methods because of their clear staining background, the sensitivity to staining condition. But there were breed differences and tissue specificity in the optimal preincubation condition. The optimal acid preincubation condition for classifying muscle fibers was pH4.30 for LT, while pH 4.35 for the LS and LR pigs. Meanwhile the optimal acid preincubation condition was pH4.35 for PM, while pH4.40 for TM or SM. For further selection from 2, 3, 4-AC, in-situ RT-PCR was applied to detect the mRNA distribution of myosin heavy chain I (MyHC-I). By combining in-situ PCR with enzyme histochemistry methods, MyHC-I gene and its product – Type I fibrocytes were directly located in cells at both molecular level and morphological level. Compared with the cross-sectional area (CSA) of different muscle fibers (i.e. I, II, βR, αR, αW, IIa, IIx and IIb) identified by enzyme histochemistry, it was found that the CSAs with stronger mRNA expression signal of MyHC-Ⅰ were closer to those of the Type I muscle fiber measured by 4-AC enzyme histochemistry (*P* > 0.05). Therefore, 4-AC may be considered as the most proper muscle typing method to study muscle fiber typing as well as meat quality. And the combination of in-situ RT-PCR and histochemistry may help better understand porcine muscle fiber characteristics and meat quality in pigs.

## Introduction

Muscle fibers characteristics are usually considered as a critical criteria for pig industry (England et al., 2013; Listrat et al., 2016) because they are closely related with meat quality, carcass trait, and growth performance in livestock and poultry (Kokoszyński et al., 2019; Ozawa et al., 2000). Muscle fiber characteristics largely depend on muscle fiber types (Song et al., 2020), which directly affect meat tenderness (Hwang et al., 2010; Roy et al., 2018), intramuscular fat content (Bravo-Lamas et al., 2018), the color and chroma of meat (Purslow et al., 2019), pH value, system hydraulic power, drip loss and juiciness (Huo et al., 2021). Muscle fibers can be classified according to their contractile and metabolic properties (Lefaucheur et al., 2011). Different types of muscle fibers exist different biochemical and biophysical characteristics such as oxidative and glycolytic capacities, contraction speed, myoglobin, and glycogen content (Kim et al., 2018), among which the oxidation or reduction of myoglobin can affect pork color and chroma (Mancini, 2009; Suman & Nair, 2017).

Studies indicated that various fiber typing methods are dependent on different staining methods (Dubowitz & Pearse, 1960; English & Weeks, 1984). For instance, muscle fibers are classified into oxidative (I) and glycolytic type (II) (Klont et al., 1998) according to their AM-ATPase stability to acid or alkali solutions (Karlsson et al., 1999) or red and white muscle fiber by electrostimulation (Ranvier, 1873). Also, muscle fibers could be classified into slow-twitch oxidative (SO), fast-twitch oxidative glycolytic (FOG) or fast-twitch glycolytic (FG) based on the activities of AM-ATPase and oxidative enzymes (Davis, 1986); they are also classified into slow-twitch red (βR), fast-twitch red (αR) or fast-twitch white (αW) (Ashmore & Doerr, 1971; Dai et al., 2009) or oxidative type (I), intermediate type (IIa), or glycolytic type (IIb) (Ishamri & Joo, 2017; Ryu et al., 2019) based on the quantity of their mitochondria. Besides, muscle fibers could be classified into slow-twitch oxidative (slow/I), fast-twitch oxidative glycolytic (IIa), fast-twitch glycolytic (IIb) and intermediate fast-twitch oxidative glycolytic (IIx/IId) (Zeng et al., 2020) according to their different characteristics of myofibril contraction and metabolic types (Karlsson et al., 1999) expressed in pig skeletal muscle (Lefaucheur et al., 2002), The results of muscle fiber typing vary according to different classification criteria (Lefaucheur et al., 2004), animal species (Solomon et al., 1988), breeds (Guo et al., 2011), and growth stage (Cooper et al., 1970). Also, the transformation of muscle fiber types varies according to genetic (Zhang et al., 2019) and nutritional factors i.e. arginine (Chen et al., 2018) and linoleic acid (Lu et al., 2017).

These methods above could directly reflect muscle fiber characteristics, but to some extent confused the naming and classification of muscle fibers. Current studies about muscle fiber typing mainly focused on its impact on meat quality (Chang et al., 2003). Few research focused on the variation among different fiber typing methods. To address these problems and explore the difference and correlation of different histochemical methods, 6 histochemical methods were conducted in this experiment to differentiate fiber types into 2 types of fibers (I and II), 3 types (βR, αR and αW) or 4 types (I, IIa, IIx and IIb). Moreover, in-situ RT-PCR was applied to directly localize the gene of MyHC-I in cells, which could correspond MyHC-Ⅰ genes to their products at the molecular and morphological level.

## Materials and methods

### Animal care

All animal care and handling were approved by the Ethics Committee for Animal Experimentation, South China Agricultural University. The protocols were supported by the regulations for animal experiments established by the Ministry of Science and Technology in China (2014).

### Animals, diets, and experimental design

A total of 72 healthy castrated male pigs involved in three different porcine breeds (24 pigs per breed) were used in this study: Large Spotted (LS) at d 120 ± 1, Lantang (LT) at d 180±1 and Landrace (LR) pigs at d 120 ± 1. LR was lean pigs from Danish, while LS and LT were Chinese local pigs. 24 pigs per breed were randomly divided into 4 groups according to body weight with 6 replicates per group and 1 pig per replicate. All pigs were fed in a single pen for 30 days until their respective market age. The temperature of the pig house is controlled at 26–28 °C, and the pigs can feed and drink freely. Other feeding management procedures shall be carried out according to the routine management requirements of pig farms. The basal diet was prepared according to the nutrition requirement standard (GB/T39235-2020 Nutrient requirements of pigs).

Muscle fiber samples, including psoas (PM), semitendinosus (SM) and trapezius muscle (TM), were collected from Large Spotted (LS), Lantang (LT) and Landrace (LR) pigs at their market-ages (LS at 150 d, LT at 210 d, and LR at 150 d). 6 castrated male pigs were per breed. LR was lean pigs from Danish, while LS and LT were Chinese local pigs. Within 45 min after postmortem, samples of PM, SM, and TM were obtained from the left side of the carcass. They were cut into 0.5 × 1.0 × 1.0 centimeter sizes and rapidly stored in liquid nitrogen until analyzed. Transverse muscle sections (10 µm) were obtained from each frozen muscle sample at −30°C with a frozen sectioning machine (Leica, Germany) and mounted on glass slides.

### Data and sample collection

Slices of the sample were stored at room temperature for 30 min for securing before preincubation. Preincubation was performed under different acid preincubation conditions (pH 4.20, 4.25, 4.30, 4.35, 4.40, 4.45, 4.50, 4.55 and 4.60) (Solomon et al., 1988) or alkali preincubation conditions (pH 10.20, 10.25, 10.30, 10.35 and 10.4). Then the activities of Myosin AM-ATPase and SDH were detected by using 6 kinds of histochemical methods, including acid two-fiber (2-AC), alkaline two-fiber classification(2-AL), 3-AC, 3-AL, 4-AC, and 4-AL. The muscle fibers were divided into Type I and II according to the nomenclature of Brooke & Kaiser (1970); the muscle fibers were divided into βR, αR and αW according to the nomenclature of Karlsson et al. (1993); the muscle fibers were divided into Types I, IIa, IIx and IIb according to the nomenclature of Delp & Duan (1996).

Direct in-situ reverse transcription polymerase chain reaction (in-situ PCR) was performed directly on the frozen section. By in-situ PCR, MyHC-I gene and its product - Type I fibrocytes were directly located in cells according to the modified methods of Nuovo (2006). Primer 5.0 was used to design primers for in-situ RT-PCR detection of MyHC-Ⅰ based on porcine sequence(Accession No. AY390526). Primer parameters were shown in Table 1. The amplified fragment length was 116 bp. Biotin (Sangon, Shanghai) was applied to the 5′ end of the upstream primer.

**Table 1:** Primers and runs of real-time RT-PCR.

Target gene	Primer sequence	Amplification product (bp)	Annealing temperature Tm( °C)
MyHC-slow/I (AB053226Genbank)	F: 5′-GAGAAGGGCAAAGGCAAGG-3′	116	59°C
	R: 5′-ACGAAGTGGGGATGTGTGG-3′		

The relative abundance of MyHC-I mRNA was analyzed by a GeneAmp In-situ PCR System (LongGene, Hangzhou, China). Reverse transcription for in situ PCR was performed using 10 μL 5 × MMLV - Buffer, 4 μL dNTP (10 mM), 3 μL N_10_ Primers (10 mM), 0.3μL RNase Inhibitor, 1 μL MMLV(200 U/μL)and 31.7 μL RNase free ddH_2_O. The following schemes are adopted:(i) denaturation procedure (5 min at 94°C); (ii) Amplification and quantification procedures were repeated for 30 cycles (94°C 1 min, 58°C 1 min, 72°C1min) and 72°C 7 min. The samples were washed with RNase-free ddH2O for 5 min and gradually dehydrated with 50 %, 75 % and 100 % ethanol. ddH_2_O was used instead of reverse transcription reagent for negative control.

### Statistical analysis

Motic imaging system (Motic China Group Co. Ltd) was used to take pictures of different fields of the section, and the pictures taken at 400 times magnification were selected for determination and analysis. The area of DAB-colored spots in muscle fiber cells and cross-sectional area (CSA) of the muscle fiber cells were determined. Calculate the average area of at least 50 MyHC-I fibers. The number percentage of muscle fibers was obtained by the ratio of the number of each fiber type to the total number of fibers counted; The number density of each fiber is the number of fibers per unit area. All data were analyzed by SPSS 23.0 statistical software. The differences of CSA in different groups of pigs were compared by single factor ANOVA. Multiple comparative analysis was performed by LSD program, and the results were expressed as mean ± standard error, *P* < 0.05 means significant difference.

## Results and discussion

**Fig. 1:**
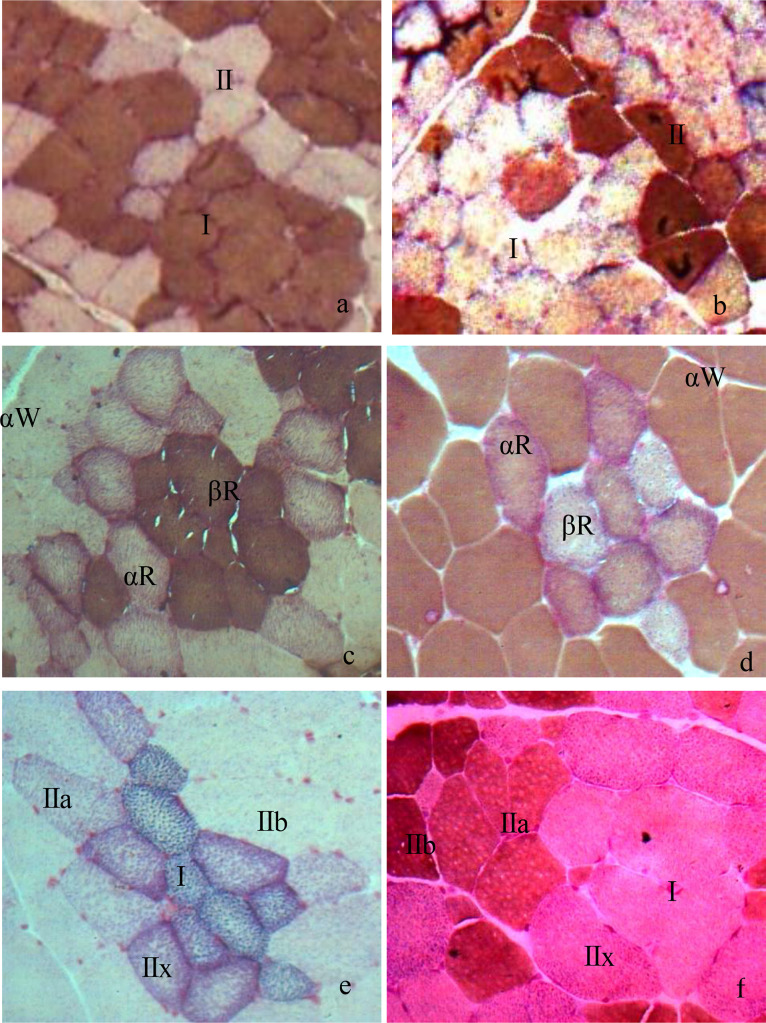
Histological images of skeletal muscle fibers types in 6 different muscle-fiber classification in swine (10 × 40). Note: a. Two types of fibers (I and II) were differentiated by acid two-fiber (2-AC); b. Two types of fibers (I and II) were differentiated by alkaline two-fiber classification (2-AL); c. three types of fibers (βR, αR and αW) by acid three-fiber classification (3-AC); d. three types of fibers (βR, αR and αW) by alkaline three-fiber classification (3-AL); e. four types of fibers (I, IIa, IIx and IIb) by acid four-fiber classification (4-AC); f. four types of fibers (I, IIa, IIx and IIb) by alkaline four-fiber classification (4-AL).

Pigs with top meat quality are usually selected based on their growth performance, carcass composition, and meat quality traits (Kim et al., 2018; Listrat et al., 2016), which are closely related to muscle contraction, metabolic characteristics (Park et al., 2009), and muscle fiber composition (Zhang et al., 2019). Muscle fibers can be classified into different types (Dubowitz & Pearse, 1960; English & Weeks, 1984), each of which has different biochemical and biophysical properties, such as oxidative and glycolytic capacities, contraction speed, myoglobin, and glycogen content (Kim et al., 2008). In general, differences in sensitivity of the AM-ATPase activity to pH preincubation have been widely used to classify muscle fibers (Guth & Samaha, 1970). For instance, 3 types of muscle fibers in pigs (types I, IIA, and IIB) can be distinguished after preincubation at pH4.35 (Lefaucher et al., 1991). The different metabolic types of pig muscle fibers can be distinguished by changes in the activity of SDH, such as being divided into oxidized (red) and non-oxidized (white) fibers (Gauthier, 1969). These results were similar with those of acid muscle-fiber classification in this study (Karlsson et al., 1999; Karlsson et al., 1993). In this study, 6 kinds of histochemical methods combining AM-ATPase with SDH were conducted to differentiate fiber types. Two types of fibers (I and II) were differentiated by acid two-fiber (2-AC) or alkaline two-fiber classification(2-AL), three types of fibers (βR, αR and αW) by acid three-fiber (3-AC) or alkaline three-fiber classification(3-AL), and four types of fibers (I, IIa, IIx and IIb) by acid four-fiber classification (4-AC), or alkaline four-fiber classification (4-AL)(Fig. 1). In 2-AC method, the color of type I in muscle fibers is the darkest, while the color of type II is the lightest (Ashmore & Doerr, 1971; Dutson et al., 1971; Solomon et al., 1990); in 3-AC, the color of βR is the darkest while the color of αW is the lightest (Delp & Duan, 1996; SuzukiA, 1980). However, the color of each muscle fiber type in the alkaline acid muscle-fiber classification was just opposite to that in the acidic classification (Fig. 1).

**Fig. 2:**
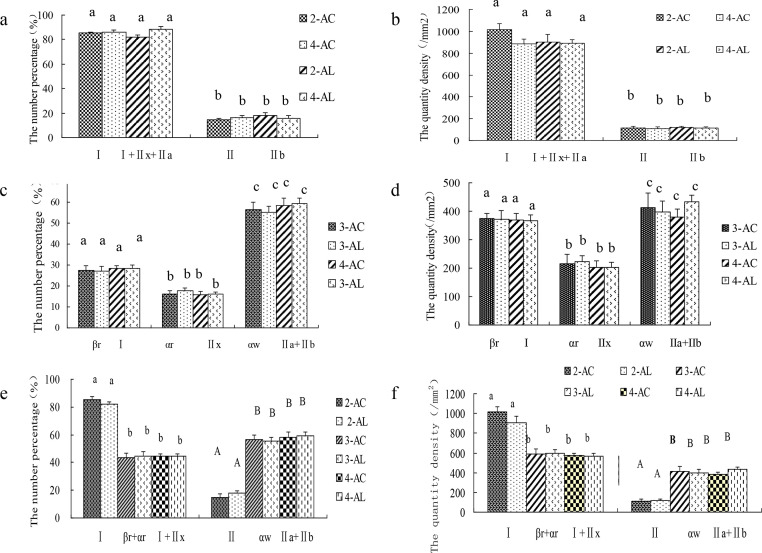
The muscle fiber characteristics difference of the psoas muscle (PM) in large spotted pigs in different muscle-fiber classification (*n* = 6) (a) The number percentage of different types of muscle fibers in 2-AC, 2-AL, 4-AC and 4-AL muscle-fiber classification; (b) The quantity density of different types of muscle fibers in 2-AC, 2-AL, 4-AC and 4-AL muscle-fiber classification; (c) The number percentage of different types of muscle fibers in 3-AC, 3-AL, 4-AC and 4-AL muscle-fiber classification; (d) The quantity density of different types of muscle fibers in 3-AC, 3-AL, 4-AC and 4-AL muscle-fiber classification; (e) The number percentage of different types of muscle fibers in 6 different muscle-fiber classification; (f) The quantity density of different types of muscle fibers in 6 different muscle-fiber classification. Note: No letters mean that there is no significant difference (*P* > 0.05); the same below.

The difference and correlation of 6 different histochemical methods were analyzed in Fig. 2. There was a degree of correlation among the 2, 3 or 4 muscle-fiber classification. The muscle-fiber number percentage of Type I in the 2-AC method is equal to the number percentage of the sum of I, IIx and IIa in the 4-AC method (Fig. 2a). Similar results were found in the quantity density between the 2-AC and 4-AC methods (Fig. 2b). The characteristics of Type II in the 2-AC method is similar with those of IIb in the 4-AC method (Fig. 2a,b). The muscle-fiber characteristics of βR in the 3-AC method is similar with those of Type I in the 4-AC method (Fig. 2c,d). The muscle-fiber characteristics of αR in the 3-AC method is similar with those of IIx in the 4-AC method (Fig. 2c, d). The muscle-fiber characteristics of αW in the 3-AC method is similar with those of IIa + IIb in the 4-AC method (Fig. 2e, f). However, there were no significant differences in the number percentage and the number density of different types of muscle fibers between acidic (AC) and alkaline (AL) classification, no matter in 2, 3 or 4 muscle-fiber classification (Fig. 2) (*P* > 0.05), that meant the results of AC and AL muscle-fiber classification were consistent in reflecting the characteristics of muscle fibers.

**Fig. 3:**
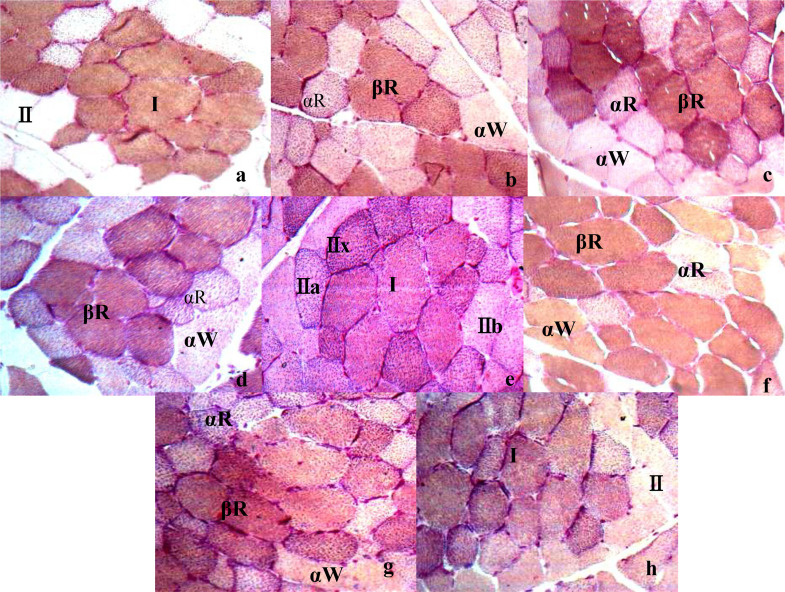
Histological images of the trapezius muscle (TM) from large spotted pigs (LS) when treated by acid preincubation solutions at different pH (10 × 40) in acid muscle-fiber classifications. Note: a–h: TM in acid preincubation solutions at pH 4.20/pH 4.25/pH 4.30/pH 4.35/pH 4.40/pH 4.45/pH 4.50/pH 4.55.

**Fig. 4:**
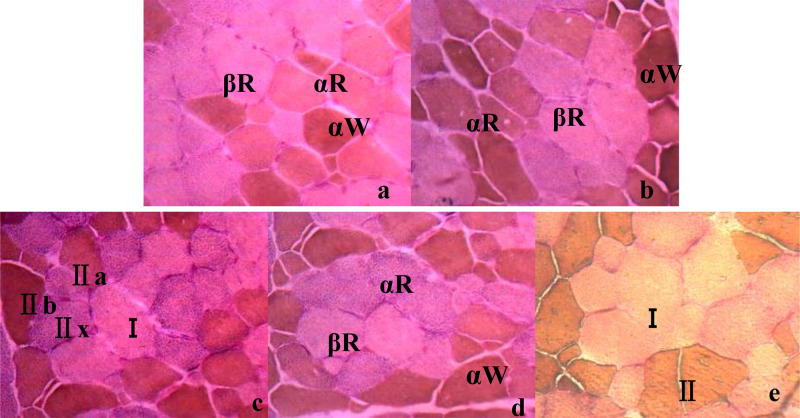
Histological image of the trapezius muscle(TM) from large spotted (LS) pigs in alkaline preincubation solutions at different pH(10 × 40) in alkaline muscle-fiber classifications. Note: a/b/c/d/e: Histological images of TM in alkaline preincubation solutions at pH 10.20/pH 10.25/pH 4.30/pH 4.35/pH 10.40.

The classification results of muscle fibers varied under different preincubation and incubation conditions(Fig. 3, Fig. 4). Under the conditions of acidic preincubation (pH4.20–4.60) and alkaline preincubation (pH 10.20–10.40), the muscle fibers in the muscle tissue were divided into 2, 3 and 4 fiber types with the change of pH. 2 muscle-fiber types (I and II) were identified at pH 4.20 or pH 4.55 under acid preincubation (Fig. 3a,h) or at pH 10.40 under alkaline preincubation(Fig. 4e). 3 muscle–fiber types (βR, αR and αW) were identified at pH 4.25–4.35 or pH 4.45–4.50 (Fig. 3b–d,f,g) under acid preincubation, or at pH 10.20–10.25 or pH 10.35 (Fig. 4a,b,d). 4 muscle-fiber types (I, IIa, IIx and IIb) were identified at pH 4.40(Fig. 3e) or at pH 10.30(Fig. 4c). Besides, by comparing AC and AL methods, AC methods may be superior to AL methods because of their clear staining background, the sensitivity to staining condition (Solomon et al., 1988). Thus, 4 types of muscle fibers can be identified at the histological level by enzymatic histochemical methods, and the optimal acid preincubation condition was pH4.35 for classifying muscle fibers in the TM of LS pigs.

**Fig. 5:**
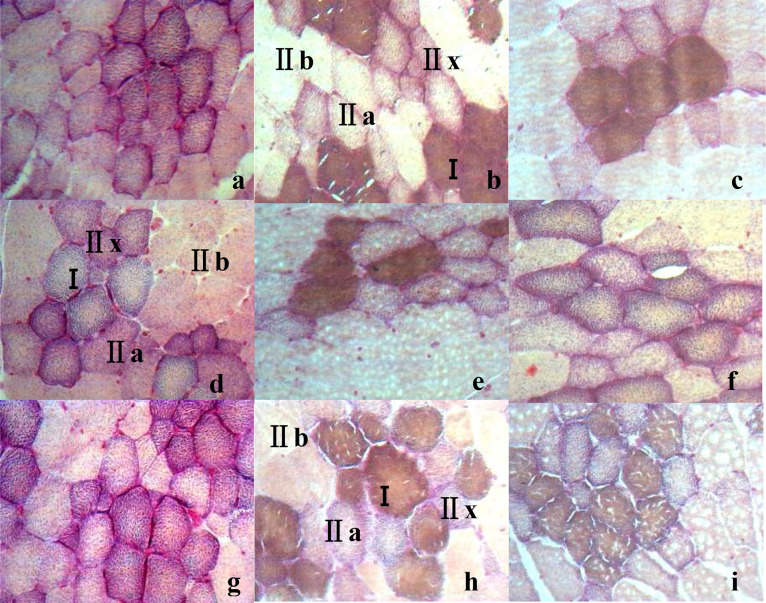
Histological images of the psoas muscle (PM) from different breeds (Large Spotted (LS), Lantang (LT) and Landrace (LR) pigs) in acid preincubation solutions at different pH (10 × 40). Note: a/b/c/d:LS at pH 4.30/ pH 4.35/ pH 4.40; d/e/f: LT at pH 4.30/ pH 4.35/ pH 4.40; g/h/i: LR at pH 4.30/ pH 4.35/ pH 4.40.

However, there were breed differences in the optimal preincubation condition (Fig. 5). Take PM for example. For Large Spotted pigs or Landrace pigs, 4 muscle-fiber types were identified just at pH 4.35 under acid preincubation (Fig. 5b,h), while 2 or 3 muscle-fiber types were identified at pH 4.30 or 4.40 (Fig. 5a,c,g,i); but for PM in Lantang pigs, 4 muscle-fiber types were identified just at pH 4.30 under acid preincubation(Fig. 5d), while 2 or 3 muscle-fiber types were identified at pH 4.35 or 4.40 (Fig. 5e,f). In comparison, the optimal acid preincubation condition for classifying muscle fibers was pH4.35 in the LS and LR pigs, while pH4.30 in LT(Fig. 5).

**Fig. 6:**
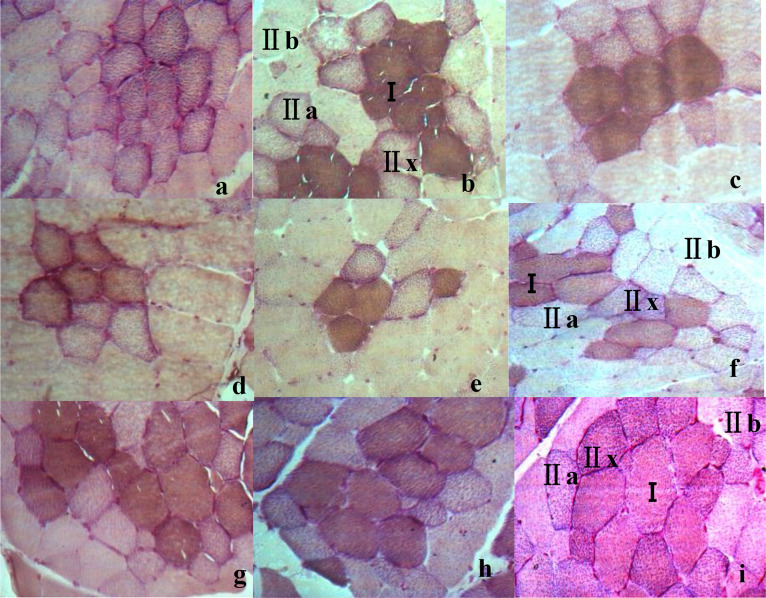
Histological images of different parts of muscle in large spotted pigs (LS) in acid preincubation solutions at different pH (10 × 40). Note: a/b/c:PM at pH 4.30/ pH 4.35/ pH 4.40; d/e/f: SM at pH 4.30/ pH 4.35/ pH 4.40; g/h/i: TM at pH 4.30/ pH 4.35/ pH 4.40.

The fiber classification results also varied with tissue differences in the optimal preincubation condition (Fig. 6). For PM in LS pigs, 4 muscle-fiber types were identified just at pH 4.35 under acid preincubation (Fig. 6b), while 2 or 3 muscle-fiber types were identified at pH 4.30 or 4.40 (Fig. 6a,c); but for TM or SM, 4 muscle-fiber types were identified just at pH 4.40 under acid preincubation (Fig. 6f,i), while 2 or 3 muscle-fiber types were identified at pH 4.30 or 4.35 (Fig. 6d,e,g,h). In comparison, the optimal acid preincubation condition for classifying muscle fibers was pH4.35 for PM, while pH4.40 for TM or SM (Fig. 6).

**Fig. 7:**
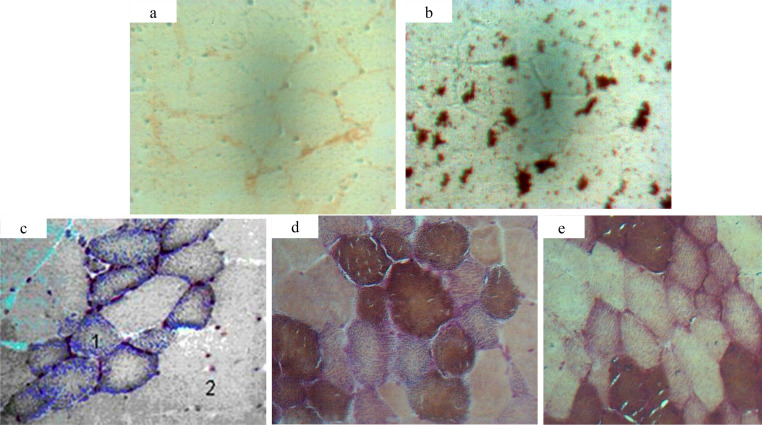
Determination of MyHC-I mRNA by in-situ RT-PCR and histological image of fiber types in porcine PM (10 × 40). (a) Negative control without RT Determination of MyHC-I mRNA in porcine PM by in-situ RT-PCR; (b) Determination of MyHC-I mRNA in porcine PM by in-situ RT-PCR; (c) Histological image of 2 types of muscle fibers in 2-AC; (d) Histological image of 3 types of muscle fibers in 3-AC; (e) Histological image of 4 types of muscle fibers in 4-AC.

In-situ RT-PCR analysis was usually used in medical science (Haase et al., 1990; Nuovo et al., 1991) for virus (Hsieh et al., 2006) and for mRNA detection (Haupt et al., 2006) at molecular and cell levels. Muscle contraction and related metabolic characteristics were determined by the expression of MyHC subtypes. Therefore, in order to further determine the accuracy of the 2, 3, 4-AC methods, in-situ RT-PCR was used to further detect the mRNA distribution of MyHC-I, which was first used to distinguish muscle fiber types. The combination of in-situ PCR and enzyme histochemistry can make up for the inaccuracy of traditional enzyme histochemistry methods (Lefaucheur et al., 2004) and the inability of fluorescence quantitative methods, which may not directly reflect muscle fiber characteristics (muscle fiber diameter, density, CSA, etc.) (Delp & Duan, 1996). By combining in-situ PCR with enzyme histochemistry, MyHC-I gene and its product - Type I fibrocytes were directly located in cells by detecting the frozen skeletal muscle sections at both molecular level and morphological level (Fig. 7b). By analyzing the difference in positive signal intensity of MyHC-I mRNA in different types of cells, results showed that the mRNA signal of MyHC-I was mainly distributed in Type I fibrocytes, which meant MyHC-I mRNA was mainly expressed in its product - Type I fibrocytes (Fig. 7b). By comparing the CSAs of cells with dense mRNA distribution of MyHC-I with those in histochemical methods, it was found that their CSAs were not significant different from those of Type I in 4-AC (*P* > 0.05)(Table 2). That meant that the 4-AC method may reflect MyHC-I genes more accurately. Therefore, 4-AC may be the most suitable method to study muscle fiber typing and meat quality. And their combination may play a vital role in exploring muscle fiber typing and improving meat quality.

**Table 2:** Comparison of the muscle fibers cross-sectional area (CSA) identified by in-situ RT-PCR and identified by different histological analysis (μm^2^) (*n* = 6).

Indexes	Histological analysis			
2-AC	Type I		Type II	
	905.67 ± 41.41^b^		1191.83 ± 45.30^a^	
3-AC	Type βR	Type αR	Type αW	
	648.08 ± 26.48^b^	545.80 ± 30.61^b^	1118.33 ± 107.17^a^	
4-AC	Type I	Type IIx	Type IIa	Type IIb
	618.51 ± 20.37^c^	520.23 ± 75.54^c^	788.90 ± 54.47^b^	1185.40 ± 61.29^a^

Note: Different superscript letters mean that there is significant difference among different muscle fiber types (*P* < 0.05); the same below.

Still, the older ATP-SDH methods have their advantages over the more modern MyHC methods. For instance, they are easier to operate and they cost less, including no need to buy more expensive in situ PCR system. So the older ATP-SDH methods were necessary and still used in the industry widely and routinely.

## Conclusion

There was a degree of difference and correlation among the 6 muscle-fiber classification. AC and AL muscle-fiber classification were consistent in reflecting the characteristics of muscle fibers, but the color of each muscle fiber type was just opposite. AC methods may be superior to AL methods because of their clear staining background, the sensitivity to staining condition. But there were breed differences and tissue specificity in the optimal preincubation condition. By combining in-situ PCR with enzyme histochemistry, MyHC-I gene and its product - Type I fibrocytes were directly located in cells at both molecular level and morphological level. And 4-AC may be the most suitable method to study muscle fiber typing and meat quality. Further study will focus on what specific fiber type is associated with the best meat quality for different market or production type.

## Ethical statement

The animal study was reviewed by Animal Care and Use Committee of South China Agricultural University, China, and conducted in accordance with the approved protocol (No. LAC2022001). Written informed consent was obtained from the owners for the participation of their animals in this study.

## CRediT authorship contribution statement

**Tao Lin:** Writing – review & editing, Writing – original draft, Visualization, Validation, Supervision, Software, Resources, Project administration, Methodology, Investigation, Funding acquisition, Formal analysis, Data curation, Conceptualization. **Zhun Liu:** Validation, Resources, Investigation, Conceptualization. **Fawen Dai:** Writing – review & editing, Formal analysis, Data curation. **Hechuan Wang:** Data curation. **Jianjun Zuo:** Project administration, Funding acquisition, Formal analysis, Data curation, Conceptualization.

## Declaration of competing interest

We certify that there is no conflict of interest with any financial organization regarding the material discussed in the manuscript.
